# Evaluation of biobased carriers derived from agri-food waste for biostimulants delivery in horticulture

**DOI:** 10.1007/s00253-026-13886-y

**Published:** 2026-05-28

**Authors:** Michele Pallucchini, Francesca Mapelli, Giulia Franzoni, Daniele Carullo, Joa Patania, Nicolò De Pizzol, Antonio Ferrante, Stefano Farris, Lorenzo Vergani, Sara Borin

**Affiliations:** 1 Department of Food, Environmental and Nutritional Sciences, University of Milan, Milan, Italy; 2 Department of Agricultural and Environmental Sciences, University of Milan, Milan, Italy; 3 Department of Earth and Environmental Sciences, University of Pavia, Pavia, Italy; 4 Institute of Crop Science, Sant’ Anna School of Advanced Studies, Pisa, Italy

**Keywords:** Biostimulant carriers, Plant growth-promoting bacteria, Biobased materials, Circular economy, Lettuce, Sustainable agriculture

## Abstract

**Abstract:**

The shift toward sustainable agriculture has increased interest in biostimulants and in novel strategies for their delivery to crops. Key challenges include improving the stability, field performance, and environmental compatibility of these agents. This study investigates a circular economy approach to develop biobased carriers from cellulose and pectin for the delivery of two plant growth-promoting bacteria, *Bacillus* sp. LR01 and *Rhizobium* sp. GR12, and a phytoextract obtained from leafy vegetable by-products. Both bacterial strains exhibited distinct colonization patterns in the rhizosphere of lettuce and tomato. When incorporated into two biobased carriers (BC1 and BC2), *Rhizobium* sp. GR12 did not survive desiccation and was therefore alternatively encapsulated in alginate beads showing a survival rate of 10^8^ CFU/g of material for 28 days of storage. The spore-forming *Bacillus* sp. LR01 maintained high viability in both formulations (10^7^ to 10^9^ CFU/g) for 28 days. The silica-enriched BC2 formulation was selected for biostimulant delivery in greenhouse trials for its improved structural integrity, while alginate was chosen as benchmark. Application of all three biostimulants as aqueous suspensions significantly enhanced lettuce growth increasing leaves biomass by 20% to 30%. Delivery via the selected carriers maintained bacterial viability and sustained rhizosphere colonization (10^6^ to 10^8^ CFU/g of soil) but reduced or reversed plant growth-promotion effects. Conversely, phenol-related secondary metabolism responses were triggered only when bacteria were delivered through the carriers. Overall, biostimulant performance depended not only on successful delivery but appeared strongly related with the physiological compatibility of the carrier with the plant–soil system.

**Key points:**

• *Waste-derived biobased materials were designed for biostimulants delivery.*

• *Biostimulants provided as aqueous suspensions enhanced lettuce growth.*

• *Biobased materials maintained microbial viability but reduced plant growth promotion.*

**Supplementary Information:**

The online version contains supplementary material available at 10.1007/s00253-026-13886-y.

## Introduction

Food systems play a key role in all societies and are fundamental to sustainable development, with efficient resource use being one of the top priorities for the coming years (United Nations Development Programme 2024). Nonetheless, over recent decades, several factors have synergistically contributed to the deterioration of agricultural soils. The heavy reliance on chemical fertilizers in agriculture poses major issues in terms of energy consumption and environmental pollution, including water eutrophication and soil degradation, especially in terms of loss of fertility and biodiversity (Liu et al. 2021; Menegat et al. 2022). Overall, these issues have created an urgent need for sustainable agricultural practices that do not compromise productivity. In this context, the use of biostimulants in agriculture as complementary tools to mineral fertilization holds great promise (Khan et al. 2023). Biostimulants are defined as products that do not directly supply nutrients to crops but rather support growth and productivity by modulating plant physiology, enhancing nutrient uptake, or improving stress tolerance (du Jardin et al. 2025). They are derived from a variety of sources and may contain plant and seaweed extracts, protein hydrolysates, microorganisms, and more (Calvo et al. 2014; Yakhin et al. 2017).

Plants are a valuable source of bioactive compounds that can be extracted, concentrated, and applied to crops to exert a beneficial effect (Wazeer et al. 2024). For example, borage extracts have been successfully used as biostimulants to enhance lettuce yield and quality (Bulgari et al. 2017) and to promote nitrate assimilation in rocket (Bulgari et al. 2020). Moreover, plants naturally establish close associations with a community of microorganisms—the plant microbiota—which support plant fitness and resilience through mutualistic interactions (Bulgarelli et al. 2012). The microbiota includes beneficial microorganisms that can be isolated, characterized, and used as plant growth-promoting (PGP) crop inoculants. Microbial biostimulants, formally recognized under the EU Fertilising Products Regulation (n. 1008/2019), provide essential services such as enhancing nutrient bioavailability, stimulating seed germination, promoting root development through hormone regulation, and protecting plants against pathogens and abiotic stresses (Compant et al. 2025). To exert their beneficial effect, microbial inoculants must successfully colonize the plant and establish within the rhizosphere or plant tissues (Berg et al., 2021); therefore, biostimulant’s formulation is crucial to improve survival and functional performance of beneficial strains once applied to crops or soil (Rebelo Romão et al. 2022). In fact, establishment can be hindered by factors such as the physiological state of the cells at application and the inherent variability of soils, climates, and crop species, often resulting in inconsistent performance in field trials (Sessitsch et al. 2019). Although liquid microbial inoculants using various aqueous carriers have been widely applied due to their simplicity and ease of use, they are often limited by factors such as the need of large application volumes and short product shelf life, which can compromise their practical scalability and effectiveness (Bashan 1998). The ideal carrier should constitute a protective environment, preventing any population decline during formulation, storage, and after introduction into soil. Furthermore, the designed products should be easy-to-use, environmentally friendly, and made of cost-effective and readily available materials (Mapelli et al. 2022).

In the present work, we explored the use of two plant-derived biopolymers, such as pectin and cellulose, as carriers for the delivery of plant-extracted and microbial biostimulants in horticultural applications, hypothesizing their positive role in improving release, plant colonization, and growth-promotion effect (Carullo et al. 2024). Biobased materials have gained increased interest as they can serve not only as sustainable and stable carriers for plant growth-promoting bacteria (PGPB), but can also offer functional properties such as controlled release, moisture retention, root adherence, and protection against environmental stress, enhancing the performance of microbial inoculants in the rhizosphere, while also improving handling and shelf life (Zvinavashe et al. 2021). These carriers can be composed of biopolymers such as alginate, starch, cellulose, polyvinylpyrrolidone, and chitosan (Zvinavashe et al. 2021), that can be effectively extracted from agri-food waste raw biomass. Biodegradable materials incorporating biostimulants can then be formulated as powders, beads, or seed coatings for delivery to crops, or used to make biodegradable nursery pots offering a renewable alternative to the oil-based materials now common in floriculture and horticulture (Schettini et al. 2013; Mapelli et al. 2022). Overall, this approach offers the advantage of reducing the ecological impact of carriers’ environmental release and represents a step forward in reducing both plastic use and agrochemical inputs in agriculture in a circular economy framework (Mapelli et al. 2022).

However, the effectiveness of waste-derived, biobased materials for biostimulant delivery in agricultural production needs careful and case-specific evaluation, as biodegradability does not guarantee The aim of this study was to test two waste-derived, biobased carrier prototypes for the incorporation and delivery of two types of biostimulants: (i) a plant extract obtained from fresh-cut vegetable waste and (ii) previously characterized PGPB belonging to the genera *Bacillus* and *Rhizobium*. The selected bacterial strains were further evaluated for their ability to colonize the root system and for their viability within the two material formulations. Both types of biostimulants embedded in the biobased carriers were supplied to lettuce plants grown under greenhouse conditions to assess their effectiveness in promoting plant growth in comparison with benchmark inoculation methods (alginate beads and direct liquid inoculation).

## Materials and methods

### Bacterial strain selection and in vitro characterization

Two plant-associated bacterial strains previously isolated and characterized for their in vitro and in vivo plant growth-promoting potential (Carullo et al. 2024; Vergani et al. 2024) were selected for this study: *Bacillus* sp. LR01, isolated from the endosphere of lettuce roots, and *Rhizobium* sp. GR12, isolated from grapevine roots tissue, as described in Vergani et al. (2024).

Taxonomic identification was primarily based on 16S rRNA gene partial sequencing and confirmed by genome sequencing for strain GR12, as previously described (Vergani et al. 2024). For *Bacillus* sp. strain LR01, identity confirmation was achieved through sequencing of the *gyr*B gene (Eurofins Genomics, Germany), following the protocol of Yamamoto and Harayama (Yamamoto & Harayama 1995).

Strains were evaluated for growth under a range of abiotic stress conditions and for their polysaccharide degradation capabilities. For thermal tolerance, single colonies were streaked on tryptic soy agar (TSA) and incubated at 4, 15, 25, 30, and 37 °C for 10 days. Tolerance to acidic conditions was evaluated by streaking the strains on TSA medium with pH values adjusted to 5 and 6. Salt and osmotic stress tolerance were assessed in tryptic soy broth (TSB) supplemented with NaCl at 2, 4, and 6% (w/V), and with polyethylene glycol (PEG) 6000 at 15, 20, and 25% (w/V). Polysaccharide hydrolytic activity was determined qualitatively following the methodology of Barbato et al. (2022). Briefly, strains were inoculated on TSA medium supplemented with starch (0.5% w/V), alginate (0.2% w/V), or pectin (0.2% w/V). Agarolytic activity was tested on TSA medium (1.5% w/V agar). After inoculation, the plates were incubated at 30 °C for 3 days. Degradation halos were observed after flooding the plates with Lugol’s iodine reagent (for agarolytic activity) or Gram’s Iodine (for starch, alginate, and pectin degradation) for 5 min and subsequent rinsing.

### Generation of fluorescently tagged strains

To enable downstream re-isolation from plant roots, rifampicin-resistant mutants of both *Rhizobium* sp. GR12 and *Bacillus* sp. LR01 strains were obtained by inoculating pure cultures on TSA supplemented with rifampicin 100 µg mL^−1^ and selecting emerging resistant colonies. These mutants were used for all subsequent tagging and inoculation experiments.

For stable genomic integration of a fluorescent marker, *Rhizobium* sp. GR12 was previously labelled via Tn7-based delivery of a gfpmut3* cassette (Vergani et al. 2024). *Bacillus* sp. LR01 was transformed in this study with the plasmid pTU2-AmyE-Pveg-eGFP, encoding chloramphenicol resistance and an enhanced GFP under control of the strong constitutive *Pveg* promoter (Wicke et al. 2017). Five hundred nanogrammes of plasmid was electroporated in 80 µL of electrocompetent cells (10^8^ cells) in a 1-mm cuvette at 2.1 kV (5 ms). Electrocompetent *Bacillus* sp. LR01 cells were prepared as in Meddeb-Mouelhi et al. (2012) without the addition of GLYB. After electroporation cells were recovered in 1 mL of Luria-Bertani (LB) medium supplemented with 0.5 M sorbitol and 0.38 M mannitol, incubated at 37 °C for 3 h under shaking at 200 rpm, and plated on TSA containing 15 µg mL^−1^ chloramphenicol for selection of transformants.

### Evaluation of rhizosphere colonization capability

Ten-day-old *Lactuca sativa* var. expertise and *Solanum lycopersicum* var. Rio grande plantlets were inoculated by supplementing the soil around the root collar with bacterial suspensions of *Bacillus* sp. LR01 and *Rhizobium* sp. GR12 in sterile water (10^8^ bacterial cells per soil gramme) in 5.4 × 5.2 × 3.8 cm pots. Rhizosphere colonization was quantified after 20 days of plant growth in greenhouse (26 °C, 12 h light). One gramme of rhizosphere soil was collected from each plant, serially diluted in sterile 0.9% NaCl, and plated on TSA supplemented with rifampicin and cycloheximide (100 µg mL^−1^). Plates were incubated at 30 °C for 24–48 h, and CFUs per gramme of rhizosphere soil were enumerated.

To visualize root colonization dynamics, the two fluorescently tagged strains were inoculated in 1-week-old lettuce and tomato seedlings grown on vertical ½ MS agar plates under axenic conditions. Bacteria were directly inoculated on roots as 10 µL water droplets containing 10^6^ cells mL^−1^. Transverse root sections were hand-cut after embedding root samples in 5% agarose. Roots were stained with rhodamine B (1 µg mL^−1^) to counterstain plant tissues and imaged using a Zeiss Axio Lab.A1 fluorescence microscope. GFP-tagged bacteria were observed under fluorescein isothiocyanate (FITC) filter sets, while rhodamine B-stained root tissues were detected under tetramethylrhodamine-isothiocyanate (TRITC) settings to distinguish microbial and plant structures. Colonization dynamics were monitored over the course of 1 month.

### Preparation and mineral composition analysis of a biostimulant phytoextract from fresh-cut vegetables waste

By-products from a fresh-cut leafy vegetable company located in Lombardy (Italy) were used for the production of the biostimulant phytoextract. The waste material consisted mainly of fragments of lettuce (86%), endive (9%), chicory (4%), and radish plus carrot (1%). The raw material was sampled and collected in three different batches, then pooled together for the maceration. Plant residues were roughly homogenized, then subjected to aqueous maceration using a weight-to-volume ratio of 1:2 (w/V) in distilled water. The mixture was kept in the dark at 20 °C for 25 days, defined according to the results of previous studies (Franzoni et al 2021). After maceration, the extract was filtered first through Whatman filter paper and subsequently through 0.45-µm syringe filters to obtain the final aqueous extract. Total C and total N in the phytoextract were determined using a ThermoQuest NA1500 elemental analyser (Carlo Erba, Milano, Italy). Specifically, 50 µL of extract was added to 25 mg of Chromosorb®, weighed into tin (Sn) capsules, and crimped. The concentrations of N and C in samples were calculated from the area of their respective peaks using an atropine calibration curve. Minerals were determined on 1 mL of extract digested by a microwave digestion system (Anton Paar MULTIWAVE-ECO) in Teflon tubes filled with 9 mL of 65% HNO_3_. Then, a one-step temperature ramp (increasing to 210 °C in 10 min, maintained for 10 min) was applied, followed by a cooling time of 20 min. The mineralized samples were transferred into polypropylene tubes and diluted to 1:40 with 1.3 M HNO_3_ in MILLI-Q water. The concentration of the elements was measured by ICP-MS (Agilent 7850 ICP-MS).

### Formulation and biodegradability assessment of pectin and cellulose-based biomaterials

Two pectin and cellulose-based, film-forming formulations were developed in this work to be used as biobased biostimulant carriers and were coded as BC1 and BC2 (biobased carrier 1 and 2). BC1 was prepared as previously described (Carullo et al. 2024) by dissolving 1.5% (w/w) of pectin powder from citrus peel (Sigma-Aldrich, P9135) and 0.5% (w/w) of microfibrillated cellulose powder (supplied by the Paper and Fibre Research Institute, Trondheim, Norway) in distilled water at 90 °C until complete dissolution, followed by cooling to 25 °C and the addition of glycerol (1.5% w/w). The so-obtained BC1 solution was further subjected to the incorporation of silica (3.5% w/w) according to the sol-gel approach proposed by Fuentes-Alventosa et al. (2013) to obtain the BC2 solution. Both film-forming solutions were adjusted to pH 7 with NaOH. Fifty millilitres of each liquid BC formulation was cast into square Petri dishes (120 × 120 mm) and dried uncovered at 37 °C for 72 h until complete solidification and dehydration. Strips of each material (3 × 12 cm, 0.36 ± 0.03 g) were placed circumferentially inside plastic pots (5.4 × 5.2 × 3.8 cm) filled with commercial potting substrate. Pots were maintained for 1 month in a greenhouse at 26 °C under a 12-h light/12-h dark photoperiod and watered as needed to maintain soil moisture. The physical state of the biobased material was visually monitored periodically and documented over time as a preliminary assessment for biodegradability.

### Biostimulant incorporation into pectin-based formulations and alginate beads

The bacterial strains *Rhizobium* sp. GR12 (vegetative cells) and *Bacillus* sp. LR01 (vegetative cells and spores) were incorporated into the BC1 and BC2 biomaterials. Each strain was grown for 24 h in TSB to the exponential phase, then washed twice by centrifugation (4000 × g, 10 min) and resuspension in physiological solution (0.9% NaCl). Following quantification through a Thoma counting chamber under a phase-contrast microscope, cells were resuspended in 1.5 mL of sterile distilled water and incorporated into 48.5 mL of liquid biomaterial by stirring for 5 min at room temperature to a final concentration of 10^8^ CFU mL^−1^. Fifty millilitres of each inoculated solution was poured into square 12 × 12 cm Petri dishes (3.5 × 10^7^ CFU cm^−2^, corresponding to 0.35 mL cm^−2^) and dried uncovered at 37 °C for 72 h until complete dehydration, generating solid films. The dried films contain a theoretical concentration (i.e. without considering the loss of viability during the desiccation process) of 3.5 × 10^9^ CFU g^−1^. Negative control films were prepared by adding 1.5 mL of sterile distilled water to 48.5 mL of liquid biomaterial in place of the bacterial suspension. Induction of LR01 sporulation and spore harvesting were adapted from the protocol described by Coroller et al. (2001) by incubating the strain for 7 days at 37 °C under agitation in TSB supplemented with MnSO₄ (40 mg L^−1^) and CaCl₂ (100 mg L^−1^). Spore formation was confirmed by phase-contrast microscopy at ×100 magnification. Spore suspensions were washed three times with sterile distilled water, resuspended in 50% ethanol, and incubated at 4 °C for 12 h to eliminate any residual vegetative cells. After three additional washes with distilled water, spores were enumerated using a Thoma counting chamber under phase-contrast microscopy and stored at 4 °C in sterile water. Spore viability was confirmed by plating serial dilutions on TSA and incubating at 30 °C for 24 h. A final concentration of 10^8^ spores per mL of biomaterial was incorporated into the liquid formulations, cast, and dried as described above for vegetative cells.

*Rhizobium* sp. GR12 and *Bacillus* sp. LR01 were encapsulated in alginate by resuspending the vegetative cells in a 2% (w/V) sodium alginate solution (alginic acid sodium salt from brown algae, BioReagent grade, Merck) to a final concentration of 4.5 × 10^8^ CFU per mL of beads, corresponding to a theoretical concentration (i.e. without considering the loss of viability during the spherification process) of 10^7^ CFU per bead or 10^9^ CFU g^−1^. Beads were formed by peristaltic extrusion of the alginate-cell suspension (Watson Marlow 205S peristaltic pump, 25 rpm) into sterile 100 mM CaCl₂ under continuous stirring. The resulting beads were stored at 4 °C in 100 mM CaCl₂.

The phytoextract obtained from lettuce leaves was incorporated into the BC2 biopolymer at a concentration of 10 mL L^−1^ V/V by substituting it to water during the biomaterial formulation and was desiccated at 37 °C after casting in square Perti dishes as described above.

### Viability assessment of biobased carrier-incorporated bacteria

The viability of the bacterial strains was evaluated at four timepoints after incorporation into the biobased carriers and storage at room temperature (7, 14, 21, and 28 days; D07–D28). For BC1 and BC2 films, viability of both *Rhizobium* sp. GR12 and *Bacillus* sp. LR01 was preliminarily verified by depositing 1 cm^2^ of the film on TSA plates supplemented with rifampicin (100 µg mL^−1^) and cycloheximide (100 µg mL^−1^) to selectively recover rifampicin-resistant bacterial strains and inhibit fungal growth, respectively. Plates were incubated at 30 °C for 72 h, and bacterial growth at the film–agar interface was recorded. For the quantification of viable bacterial cells, 1 cm^2^ of film (weighing 0.01 ± 0.001 g) was aseptically excised and transferred to a sterile 2-mL microcentrifuge tube containing a stainless-steel sterile bead and 1 mL sterile 0.9% NaCl solution. Samples were disrupted using a TissueLyser (QIAGEN) at 15 Hz for 2 × 1 min cycles, inverting tube racks between cycles to ensure uniform mixing. Aliquots of serial decimal dilutions were plated in triplicate onto TSA supplemented with rifampicin and cycloheximide, and CFUs were enumerated after incubation at 30 °C for 24 h.

For the quantification of viable cells of *Rhizobium* sp. GR12 after the incorporation into alginate beads, 10 beads (0.1 ± 0.01 g) were suspended in 10 mL of 100 mM phosphate buffer (pH 7.0), gently agitated for 30 min to dissolve the matrix, serially diluted, and plated on selective TSA as described above.

### *In planta* application of biostimulants via biobased carriers

The performance of biobased carriers for *Bacillus* sp. LR01, *Rhizobium* sp. GR12, and phytoextract was assessed in three independent greenhouse experiments using *L.* *sativa* var. expertise as a host plant. Treatments included either (i) direct application of aqueous inocula or (ii) delivery via biobased carriers.

Ten experimental treatments summarized in Supplementary Table 1 were applied, each replicated across ten biological replicates (plants). Four treatments evaluated the performance of the biobased carriers: (1) *Bacillus* sp. LR01 spores embedded in BC2 film (12 × 3 cm BC2 film strips containing a theoretical concentration of 10^10^ spores, corresponding to a measured concentration of 10^8^ cells per pot); (2) *Bacillus* sp. LR01 spores encapsulated in alginate beads (1 g of beads containing a theoretical concentration of 10^9^ cells, corresponding to a measured concentration of 10^8^ cells per pot); (3) *Rhizobium* sp. GR12 cells encapsulated in alginate beads (1 g of beads containing a theoretical concentration of 10^9^ cells, corresponding to a measured concentration of 10^8^ cells per pot); (4) phytoextract incorporated into BC2 film (one 12 × 3 cm strips containing 10 mL of phytoextract L^−1^ v/v). Three treatments consisted of the biostimulants applied in liquid form without any carrier material: (5) *Rhizobium* sp. GR12 cells (10⁸ cell mL^−1^) as delivery control for *Rhizobium* sp. GR12; (6) *Bacillus* sp. LR01 spores (10⁸ cell mL^−1^) as delivery control for *Bacillus* sp. LR01; (7) phytoextract (10 mL L^−1^ v/v). Three additional treatments served as negative controls: (8) water (absolute negative control); (9) uninoculated BC2 film (biobased material control); (10) uninoculated alginate beads (biobased material control). The theoretical bacterial concentrations in the alginate and BC2 carriers reported above account for the use of a higher initial inoculum to compensate for the viability loss observed during the spherification of alginate beads and desiccation of the BC2 biocarrier.

For aqueous treatments, spore suspensions of *Bacillus* sp. LR01, cell suspension of *Rhizobium* sp. GR12, and the phytoextract were pipetted directly into the soil immediately after seed sowing. For carrier-based treatments, strips of BC2 were placed circumferentially inside the pots, while alginate beads were mixed into the soil before sowing. Lettuce seeds were sown in commercial peat-based substrate (Ortaggi Supernutriente, Vigorplant srl, Italy) in plastic pots (5.4 × 5.2 × 3.8 cm) and maintained under greenhouse conditions for 25 days before sampling and recording plant growth parameters. Plants were watered regularly with tap water. To assess rhizosphere colonization, bacterial isolation and quantification were performed as described in section “Evaluation of rhizosphere colonization capability”.

### Assessment of plant growth and physiological parameters

Plant growth was assessed through both destructive and non-destructive methods. Biomass and morphological parameters were assessed measuring shoot fresh weight and length, respectively.

Chlorophyll a and b were extracted from lettuce leaves using 99.9% (v/v) methanol. Leaf discs (30 mg), obtained with a 5 mm cork borer, were incubated in darkness at 4 °C for 24 h in 15-mL tubes containing 5 mL methanol. Chlorophylls content was determined spectrophotometrically (Evolution 300, Thermo Electron Corporation) by measuring absorbance at 665.2 nm and 652.4 nm. Concentrations were calculated according to Lichtenthaler (1987) and expressed on a fresh weight (FW) basis. Total phenols were extracted with 3 mL of methanol acidified with 1% HCl from leaf discs (30 mg) incubated in darkness at 4 °C for 24 h. Absorbance was recorded at 320 nm using the same spectrophotometer. The phenolic index was expressed as Abs320 nm g^−1^ FW.

For non-destructive measurements, physiological parameters were recorded in vivo on plant leaves using a Multi Pigment Meter MPM-100 (ADC Bioscientific) to quantify chlorophyll and flavonol indexes, calculated as log(T850/T720-1) and log(F660/F375), respectively. The nitrogen-flavonol index is calculated by the MPM-100 sensor as the ratio between chlorophyll index and flavonol index.

### Statistical analyses

The three greenhouse trials were conducted independently at different times and were therefore considered experimental blocks. Because absolute values of plant physiological parameters varied among experiments due to environmental and batch-related factors (uncontrolled greenhouse fluctuations such as microclimate conditions and substrate variability), in order to account for block-to-block heterogeneity while preserving relative treatment effects, data were subjected to percent control transformation to be normalized within each experiment prior to statistical analysis. Specifically, individual replicate values for each treatment were expressed as percentage variation relative to the mean of the corresponding control group within the same experiment. This was calculated by dividing each replicate by the mean of the negative control (NC) of that experiment and subtracting 1, thereby setting the NC to 0% (Li et al. 2022). This normalization allows treatment effects to be expressed relative to the internal control of each experimental block, thereby reducing between-experiment heterogeneity in absolute values while preserving relative treatment effects (Hedges & Olkin 1985; Hedges et al. 1999). Following normalization, datasets from the three experiments were pooled and analyzed on a common relative scale. Statistical analyses were performed on these normalized values using R software. Data normality was assessed using the Shapiro–Wilk test. For normally distributed data, differences among treatments were tested using one-way ANOVA followed by Tukey’s HSD post hoc test. When the normality assumption was not met, the Kruskal–Wallis test was applied, followed by Dunn’s post hoc comparison. In all analyses, the significance level was set at *P* < 0.05.

## Results

### Selection of two biostimulant bacterial strains and evaluation of abiotic stress tolerance and polysaccharides degradation capability

Two previously isolated plant-associated bacterial strains with demonstrated PGP traits were selected for this study: *Bacillus* sp. LR01, isolated from lettuce roots, and *Rhizobium* sp. GR12, isolated from grapevine roots (Carullo et al. 2024; Vergani et al. 2024). The two strains were evaluated for their capacity to withstand a range of abiotic stress conditions (temperature, pH, osmotic, and salinity stress) and for polysaccharidase activity, relevant to their potential formulation into biobased carriers for soil application. Both strains displayed growth between 15 and 30 °C, with optimal development observed at 30 °C. Only *Rhizobium* sp. GR12 maintained visible colony formation at 4 °C, while only *Bacillus* sp. LR01 was capable of growing at 37 °C (Table 1). Both strains showed good tolerance to osmotic stress, with LR01 growing on all tested PEG 6000 levels (15%, 20%, and 25%), while *Rhizobium* sp. GR12 tolerated only up to 20% PEG concentration in the growth medium (Table 1). *Rhizobium* sp. GR12 was sensitive to saline stress at all the tested concentrations, whereas LR01 tolerated NaCl concentrations up to 6%. Both strains were able to grow under mildly acidic conditions (pH 6), but only LR01 could survive at pH 5. *Bacillus* sp. LR01 was also the only strain exhibiting polysaccharidases activity, showing the capability to degrade pectin, agarose, alginate, and starch (Table 1).

**Table 1:**
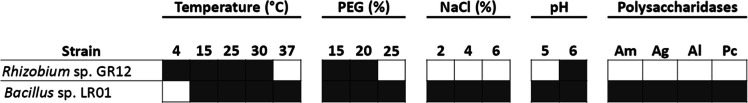
Bacterial viability under different abiotic and qualitative tests for polysaccharides degrading activities. *Am*, amylase; *Ag*, agarase; *Al*, alginase; *Pc*, pectinase. Full squares indicate bacterial growth

### *In planta* and *in vitro* assessment of rhizosphere establishment and root colonization dynamics by *Rhizobium* sp. GR12 and *Bacillus* sp. LR01 in lettuce and tomato plants

The ability of the two strains to establish in the rhizosphere of lettuce and tomato plants was preliminarily assessed in greenhouse conditions following direct soil inoculation with aqueous suspensions containing 10⁸ bacterial cells per gramme of soil. Rhizosphere colonization was evaluated by bacterial isolation at 20 days post-inoculation (dpi). Both strains were successfully isolated from the rhizosphere of lettuce and tomato seedlings, although their concentrations decreased by 2 to 6 orders of magnitude compared to the initial inoculum, with a greater reduction observed in tomato, particularly for *Bacillus* sp. LR01 (Table 2).

**Table 2:** Isolation of bacteria from rhizosphere soil. CFU per rhizosphere soil gramme at 20 dpi are reported for both lettuce and tomato plants. Significance of differences between treatments was determined by Kruskal–Wallis test followed by Dunn’s post hoc test (*P* ≤ 0.05)

	Log (CFU/g rhizosphere)	
	*Rhizobium* sp. GR12	*Bacillus* sp. LR01
Lettuce	6.53 ± 0.13^b^	7.71 ± 0.17^a^
Tomato	5.97 ± 0.24^b^	2.65 ± 0.92^a^

**Fig. 1:**
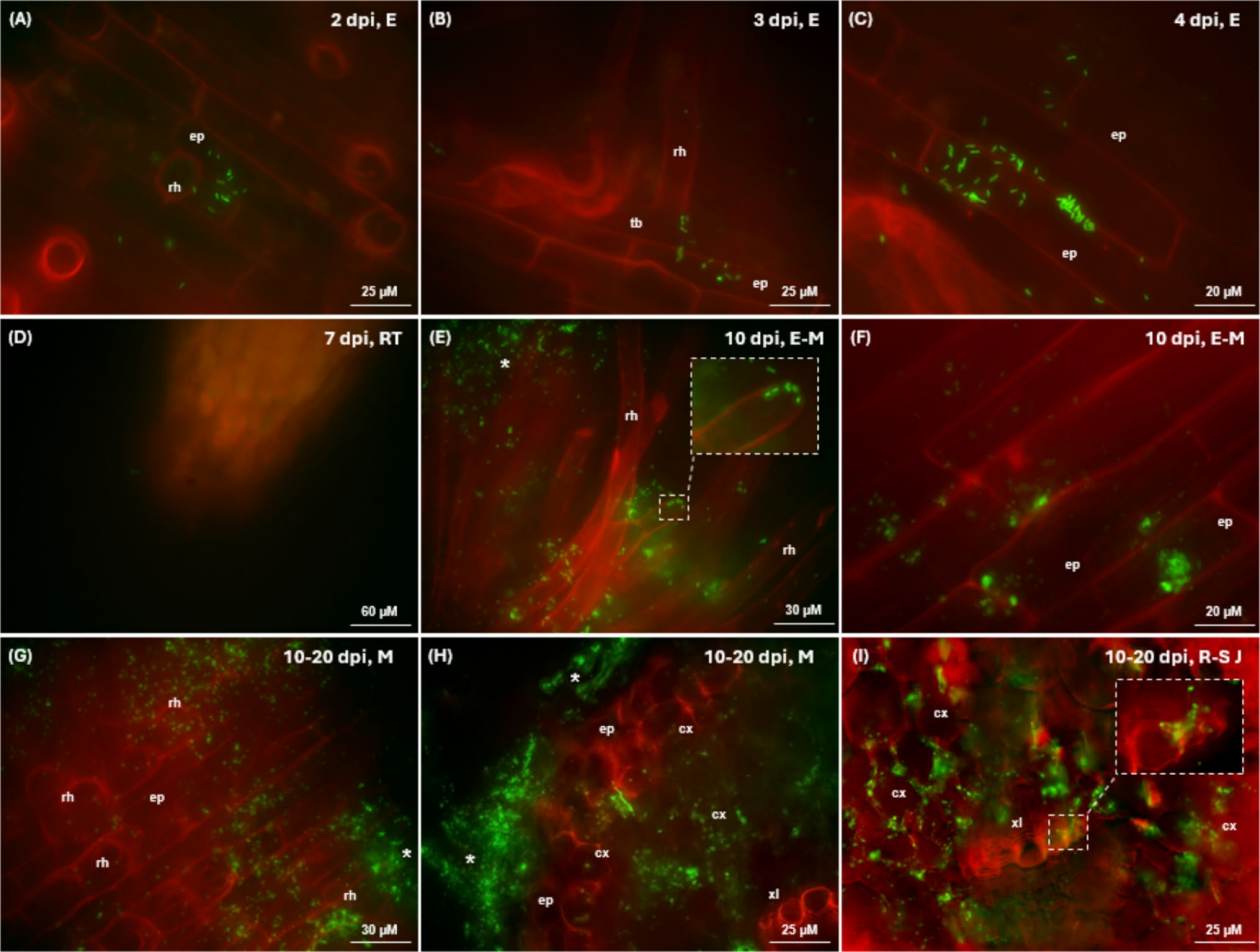
Colonization of lettuce root by fluorescently tagged *Rhizobium* sp. GR12 on MS agar, 1 to 20 dpi. Fluorescence from bacterial gfpmut3* and from plant tissues dyed with rhodamine B is visualized as green and red signals, respectively, through fluorescence microscopy. In the top right corner of each picture, the timepoint of observation (dpi) and anatomical zone of the root are indicated (E, elongation; M, maturation; RT, root tip; R-S J, root-shoot junction). **A**–**C** Individual bacterial cells colonizing epiphytically root epidermis and root hair primordia or trichoblasts in the elongation zone during early colonization stages. **D** Root tip showing no colonization. **E** Colonization of root hairs in the maturation zone, showing invasion of root hair tips. The marked root hair tip is magnified in the dashed-line inset (10×). **F** Endophytic colonization of epidermal root cells. **G** Individual bacterial cells and biofilm (white stars) aggregating on root epidermis surface and throughout root hair primordia in the maturation zone. **H** Root cross sections showing endophytic colonization of epidermis and cortex layers, with an epiphytic biofilm surrounding the epidermis (white star). **I** Root-shoot junction cross section showing endophytic colonization of the cortical parenchyma cells and xylem vessels. The marked xylem vessel is magnified in the dashed-line inset (10×). cx, cortex; ep, epidermis; rh, root hair; tb, trichoblast; xl, xylem

**Fig. 2:**
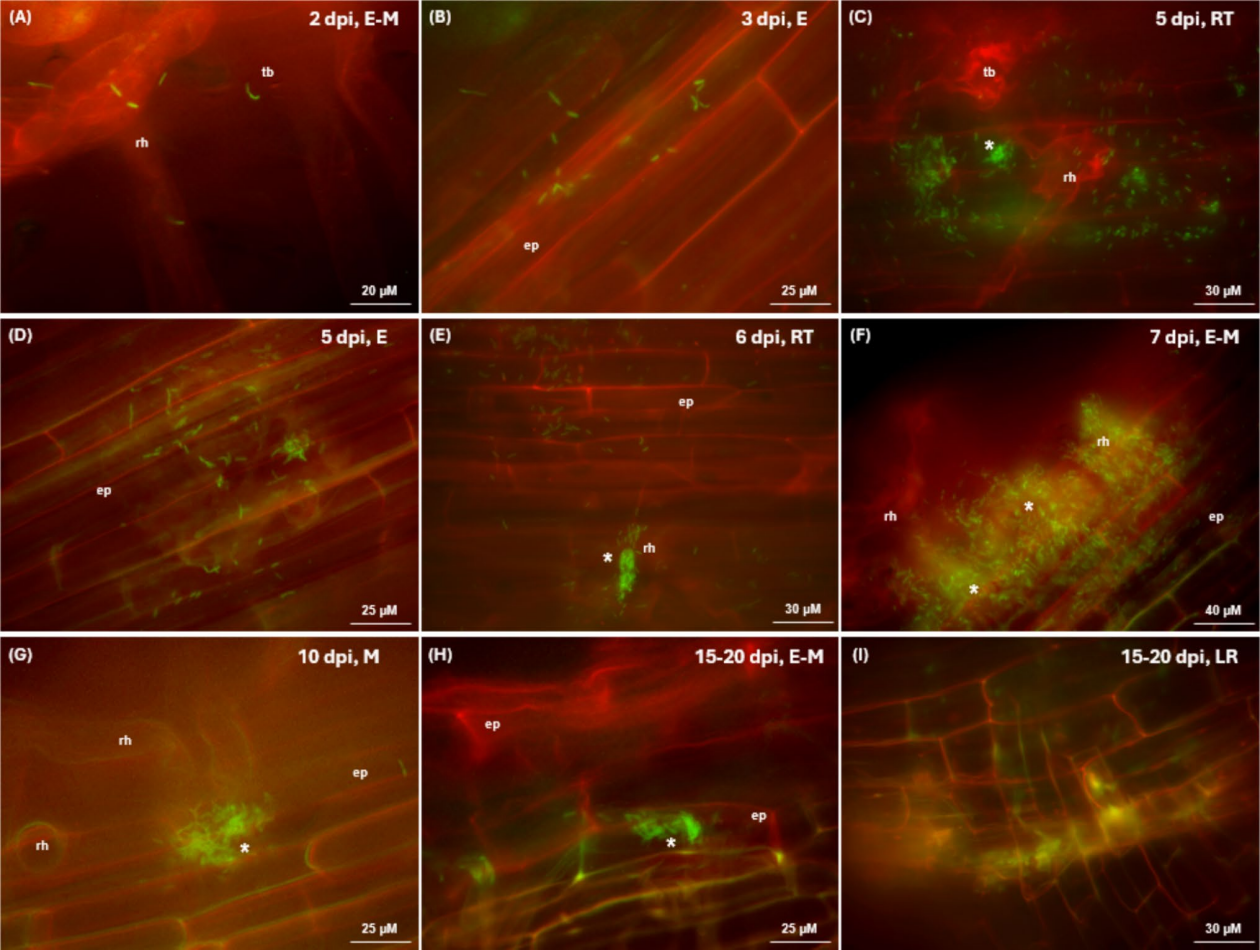
Colonization of lettuce root by fluorescently tagged *Bacillus* sp. LR01 on MS agar, 1 to 20 dpi. Fluorescence from bacterial gfpmut3* and from plant tissues dyed with rhodamine B is visualized as green and red signals, respectively, through fluorescence microscopy. In the top right corner of each picture, the timepoint of observation (dpi) and anatomical zone of the root are indicated (E, elongation; M, maturation; RT, root tip; LR, lateral root emergence site). **A**, **B** Individual bacterial cells colonizing root epidermis (**B**), root hairs, and trichoblasts (**A**) in the elongation and maturation zones during early colonization stages. **C**–**H** Epiphytic colonization of root epidermis and root hairs along the whole root length by both individual cells and large bacterial aggregates (white stars). **I** Lateral root emergence site showing no bacterial presence. ep, epidermis; rh, root hair; tb, trichoblast

To describe the colonization process, in vitro assays on ½ MS agar were conducted using GFP-labelled *Rhizobium* sp. GR12 and *Bacillus* sp. LR01 strains which were inoculated as aqueous suspensions on the seedling roots. For *Rhizobium* sp. GR12, root hairs (Fig. 1A, B, E) and epidermal cell wall junctions within the elongation and differentiation zones (Fig. 1C, F, G) served as preferred hubs for early lettuce epiphytic colonization. The strain also exhibited endophytic behaviour, as confirmed by root sectioning, which revealed colonization of cortex and xylem vessels by 5 days post-inoculation (dpi) (Fig. 1H, I). No colonization was observed at the root tip (Fig. 1D). A similar colonization dynamic was observed in tomato inoculated and imaged under the same conditions (Figure S1). Root hairs (Figure S1A) and epidermal cells (Figure S1B**, **C) were rapidly colonized epiphytically and, at later colonization stages, endophytically and possibly intracellularly (Figure S1E, F). Extensive epiphytic colonization of the epidermal layer and root hairs by individual bacterial cells and biofilms persisted and gradually increased throughout the observation period (Figure S1G, H), while xylem vessels colonization was observed during later interaction stages (Figure S1I). Unlike the lettuce model, the tomato root tip exhibited colonization, albeit to a lower extent (Figure S1D). In contrast to *Rhizobium* sp. GR12, *Bacillus* sp. LR01 displayed exclusively epiphytic behaviour (Fig. 2). It evenly distributed along the root on epidermis (Fig. 2B–D) and root hairs (Fig. 2A, E) in the elongation and maturation zones as well as close to the root tip (Fig. 2E), with no apparent preference for specific niches. Colonization by a combination of individual cells and aggregates of *Bacillus* sp. LR01 appeared static and uniform over time, with no observable progression or structural changes (Fig. 2F–H). No bacteria were observed in proximity to lateral root emergence sites (Fig. 2I).

### Formulation and characterization of a biostimulant phytoextract from fresh-cut vegetable waste

A phytoextract was produced from by-products obtained from a fresh-cut vegetable processing company located in Lombardy (Italy). The waste material consisted predominantly of lettuce residues (86%), with smaller contributions from endive (9%), chicory (4%), and radish plus carrot (1%). After 25 days of aqueous maceration at 20 °C, the extract was filtered to yield a clarified solution. The chemical composition of the phytoextract was characterized through elemental analyses. The concentration of key elements is detailed in Table 3. Potassium emerged as the predominant mineral (579.7 µg/g), followed by one magnitude order lower amounts of calcium, sodium, and magnesium. Copper and phosphorus were detected at nanogramme per gramme concentrations. The extract was found to contain 0.25% total carbon and 0.056% total nitrogen (Table 3).

**Table 3:** Content of Na, Mg, P, K, Ca, C, and N in phytoextract obtained from macerated fresh-cut lettuce waste. All values are means ± SEM (*n* = 4)

Na (µg/g)	Mg (µg/g)	K (µg/g)	Ca (µg/g)	Cu (ng/g)	P (ng/g)	C (%)	N (%)
63.6 ± 0.72	27.3 ± 0.29	579.7 ± 6.75	73.8 ± 0.64	260.4 ± 29.62	36.05 ± 0.78	0.25 ± 0.004	0.056 ± 0.005

### Development and soil persistence of two pectin and cellulose-based biomaterials

Two film-forming biobased materials, designated BC1 and BC2 (biobased carrier 1 and 2), were developed using pectin, microfibrillated cellulose, and glycerol as the primary components. The BC2 formulation was derived from BC1 through the addition of silica, introduced to enhance mechanical strength and reduce water solubility. After preparing the liquid formulations, the mixtures were cast into square Petri dishes and dried uncovered at 37 °C for 72 h. This process yielded cohesive solid films exhibiting a flexible, polymer-like consistency. To evaluate their environmental persistence, BC1 and BC2 were applied to unplanted soil in pots and incubated under greenhouse conditions for 2 weeks. Biodegradation was visually monitored over time. BC1 exhibited rapid degradation, becoming undetectable after 2 to 4 days (Figure S2A), whereas BC2 retained its structural integrity for up to 1 month (Figure S2B).

### Viability of bacterial strains into the biobased carriers

**Fig. 3:**
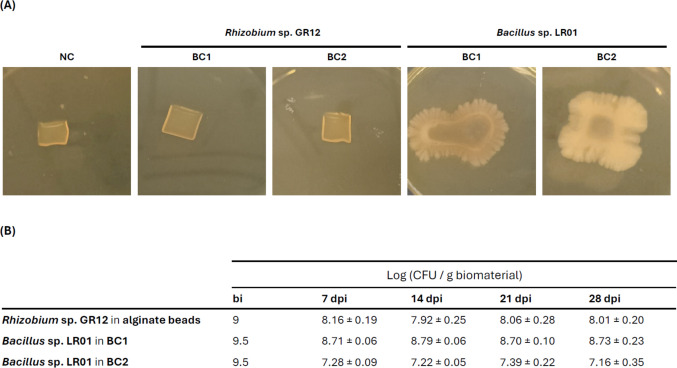
Viability assessment of bacteria incorporated into the biobased carriers. **A** Direct placement of BC1 and BC2 films carrying either water (NC, negative control), *Rhizobium* sp. GR12, and *Bacillus* sp. LR01 vegetative cells on TSA medium, showing visible growth only for strain LR01 after 5 days of incubation. **B** *Bacillus* sp. LR01 spore viability in BC1 and BC2 films and *Rhizobium* sp. GR12 viability in alginate beads over 28 days, assessed via biobased carrier disruption, dilution, and plating. Bi, before incorporation (i.e. concentration of viable cells mixed in the biomaterials before spherification or desiccation); dpi, days post incorporation

The viability of *Rhizobium* sp. GR12 and *Bacillus* sp. LR01 vegetative cells incorporated in the biomaterials BC1 and BC2 was first assessed qualitatively by placing fragments of the desiccated films on the surface of TSA plates and visually monitoring bacterial growth onto the medium. *Rhizobium* sp. GR12 showed no survival after incorporation into either biobased carrier, indicating complete loss of viability upon desiccation (Fig. 3A). Therefore, sodium alginate, a well-established bacterial biobased carrier, was selected as an alternative for downstream applications. The spore-forming *Bacillus* sp. LR01, on the other hand, remained viable for up to 4 weeks of storage at room temperature in both biobased carriers, possibly due to the survival of spores rather than vegetative cells (Fig. 3A). To enhance experimental reproducibility, *Bacillus* sp. LR01 was applied only in the spore form in subsequent incorporation experiments. The survival of *Bacillus* sp. LR01 spores in the BC1 and BC2 films and of *Rhizobium* sp. GR12 into alginate beads was quantified over 28 days of storage. The number of viable bacteria per gramme of biomaterial was determined after film dissolution in physiological solution or beads dissolution into phosphate buffer, followed by serial dilution and plating. Both strains remained viable in the tested biomaterials for 28 days at room temperature, although their survival rates differed. *Bacillus* sp. LR01 spores in BC1 showed a viability decrease of approximately one order of magnitude after 7 days, whereas in the BC2 film, the reduction exceeded two orders of magnitude. *Rhizobium* sp. GR12 vegetative cells incorporated into alginate beads exhibited a one-order-of-magnitude decrease in viability over the same period. Survival was not further reduced during the incubation period in any of the biomaterials, as cell counts remained stable after 14, 21, and 28 days of storage (Fig. 3B).

### Effect of biostimulant delivery to lettuce plants in greenhouse conditions

**Fig. 4:**
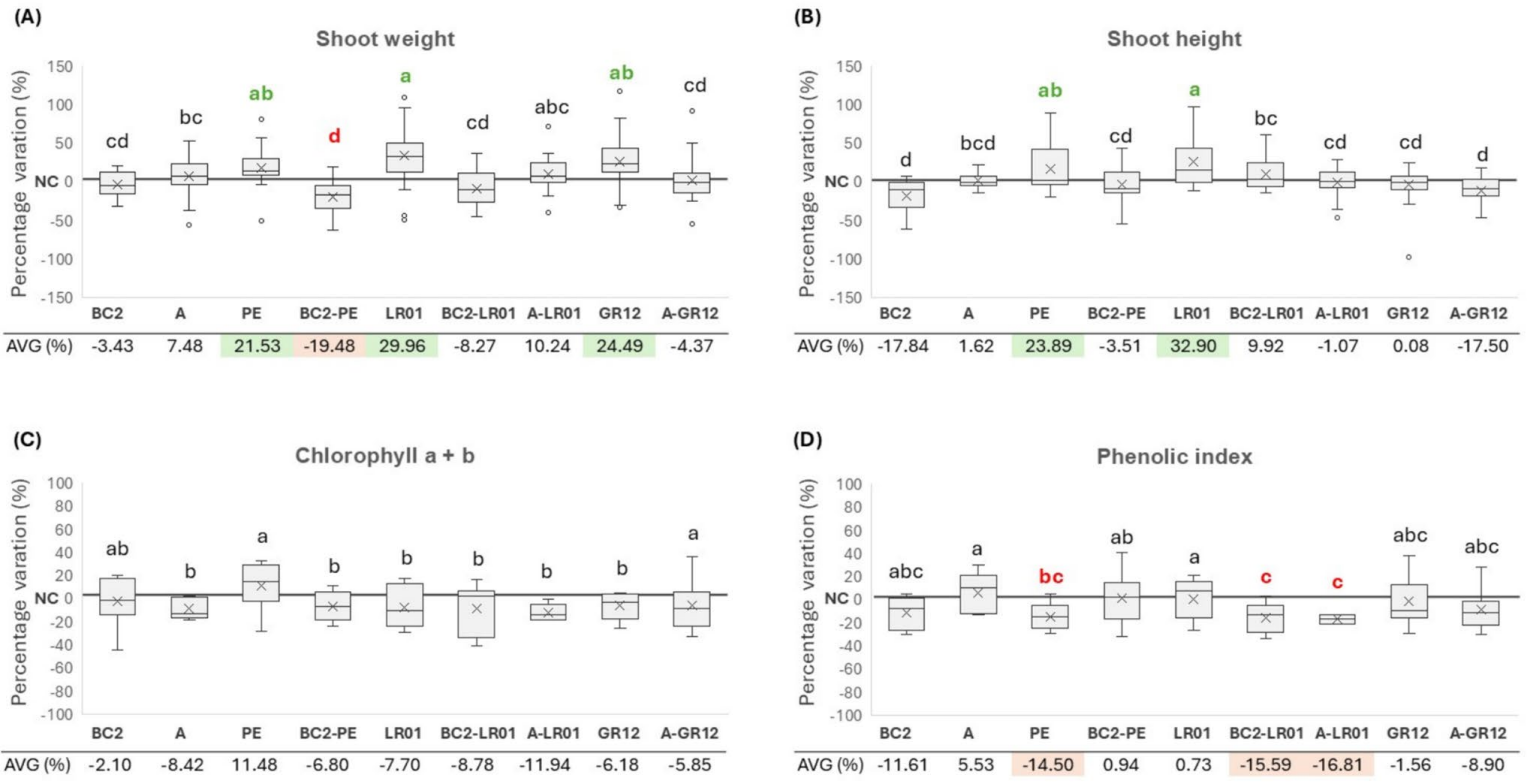
**A**–**D** Percentage variation of plant growth parameters in inoculated lettuce plants grown under greenhouse conditions. All measurements were taken 25 dpi. All values represent the percentage variation of each treatment relative to the mean of the uninoculated control (NC) within each experiment. The central horizontal line at 0% corresponds to the normalized NC baseline. Data from three independent experiments were pooled after within-experiment normalization. Statistical significance was assessed using Shapiro–Wilk tests for normality followed by one-way ANOVA with Tukey’s HSD or Kruskal–Wallis test with Dunn’s post hoc comparison when assumptions were not met (*P* ≤ 0.05). Green bold labels indicate significant increases relative to NC, whereas red bold labels indicate significant decreases. Numerical values indicating the mean percentage variation for each treatment are reported below the boxplots. **A** Shoot weight. **B** Shoot height. **C** Chlorophyll a + b content (destructive measurements). **D** Phenolic index (destructive measurements). BC2, uninoculated BC2 film; A, uninoculated alginate beads; PE, phytoextract; BC2-PE, phytoextract incorporated into BC2 film; LR01, aqueous inoculum of *Bacillus* sp. LR01 spores; BC2-LR01, *Bacillus* sp. LR01 spores incorporated into BC2 film; A-LR01, *Bacillus* sp. LR01 spores encapsulated into alginate beads; GR12, aqueous inoculum of *Rhizobium* sp. GR12 cells; A-GR12, *Rhizobium* sp. GR12 encapsulated into alginate beads

The ability of the two bacterial strains and of the phytoextract to promote lettuce growth was evaluated under greenhouse conditions across three independent experiments. To pool the results of the three experiments, all datasets underwent percent control transformation (Li et al. 2022) in order to be converted into percentage variation relative to the mean of the corresponding control group in each trial (Fig. 4). The raw data are reported in Figure S3. Treatments included either (i) direct application of biostimulants as aqueous inocula or (ii) biostimulant delivery via biobased carriers.

At 25 dpi, plant shoot height and fresh weight were measured, and chlorophyll and phenolic concentrations were assessed through destructive measurements (Fig. 4). Furthermore, non-destructive measurements were taken using the MPM-100 sensor to assess the chlorophyll content, flavonol content, and nitrogen-flavonol content indexes (Figure S4). When applied as aqueous suspensions, the phytoextract (PE), *Bacillus* sp. LR01, and *Rhizobium* sp. GR12 significantly increased shoot fresh weight relative to the control (+21.5%, +29.9%, and +24.5%, respectively), while shoot height was significantly improved only by the phytoextract (+23.9%) and *Bacillus* sp. LR01 (+32.9%) (Fig. 4A, B). None of the aqueous treatments significantly affected chlorophyll a + b content, although the phytoextract showed a non-significant increase of +11.5% (Fig. 4C). In terms of phenolic accumulation, direct phytoextract application significantly reduced the phenolic index by −14.5%, while aqueous bacterial inocula produced no significant effect. Uninoculated BC2 and alginate carriers did not induce any significant effect on plant growth, although BC2 showed a non-significant reduction of all the tested parameters and alginate non-significantly increased shoot height, weight, and phenolic index (Fig. 4A–D). As for the performance of biostimulants delivered through biobased carriers, the phytoextract embedded in BC2 (BC2-PE) caused a significant reduction in shoot fresh weight (−19.5%), reversing the positive effect observed by the aqueous application, and a non-significant reduction of shoot height (−3.5%) and chlorophyll content (−6.8%) (Fig. 4A–C). *Bacillus* sp. LR01 spores incorporated in BC2 (BC2-LR01) decreased shoot fresh weight (−8.3%) and chlorophyll content (−8.8%), although non-significantly, and significantly reduced phenolic levels by −15.6% and increased the shoot height by 9.9% (Fig. 4A–D). *Bacillus* sp. LR01 in alginate (A-LR01) increased the shoot fresh weight (+10.2%) but reduced chlorophyll content (−11.9%), both without significance, and induced a significant reduction in phenolic compounds (−16.8%) (Fig. 4A–D). Encapsulation of *Rhizobium* sp. GR12 in alginate (A-GR12) led to a non-significant reduction of shoot weight (−4.4%), height (−17.5%), chlorophyll content (−5.9%), and phenolics concentration (−8.9%) (Fig. 4A–D). Non-destructive measurements confirmed the absence of significant chlorophyll accumulation, showing minimal variation from the control in most treatments (−9.9% to +4.5%), except for A-LR01 which produced the only statistically significant change for this parameter (+22.8%) (Figure S4A). The flavonol content index showed stronger responses to BC2-based inocula, with uninoculated BC2 (+22.2%) and BC2-PE (+27.8%) both resulting in statistically significant increases, along with BC2-LR01 and A-LR01 which also showed a nonsignificant increase of +8.4% and +7.9%, respectively (Figure S4B). In contrast, the liquid application of the three biostimulants and A-LR01 induced non-significant increases ranging from −0.1% (PE) to +9.6% (LR01) (Figure S4B). For the nitrogen-flavonol index, uninoculated BC2 caused a significant decrease (−23.6%), with BC2-PE and BC2-LR01 also resulting in non-significant decreases (−22.9% and −17.9%, respectively) (Figure S4C); in contrast, A-LR01 induced a significant increase (+36.9%) and all the other aqueous or alginate-based treatments produced non-significant increases ranging from +5% (A-GR12) to +16.4% (LR01) (Figure S4C).

To assess whether the impaired PGP effect of microbial biostimulants after incorporation in the biobased carriers was the result of reduced plant colonization capability, the two bacterial strains were reisolated from plant roots at the time of sampling. A-GR12 resulted in the increase of one order of magnitude of recovered bacteria (6.61 × 10^7^ CFU per gramme of rhizosphere soil) compared to the aqueous inoculum (2.29 × 10^6^ CFU per gramme of rhizosphere soil) (Table 4). The application of *Bacillus* sp. LR01 spores resulted in the recovery of 1.41 × 10^7^ CFU per gramme of rhizosphere soil when applied as aqueous inoculum and 3.72 × 10^7^ CFU per gramme of rhizosphere soil when incorporated into alginate beads, while the number of reisolated cells decreased to 10^6^ CFU per gramme of rhizosphere soil when the spores were incorporated in the BC2 material (Table 4).

**Table 4:** Reisolation of bacteria from rhizosphere soil. CFU per rhizosphere soil gramme at 25 dpi are reported. Superscript letters indicate significance of differences between treatments, determined by one-way ANOVA test followed by Tukey’s HSD post hoc test (*P* ≤ 0.05)

	Log (CFU/g rhizosphere)	
	*Rhizobium* sp. GR12	*Bacillus* sp. LR01
Liquid inocula	6.36 ± 0.57^b^	7.15 ± 0.38^a^
Alginate beads	7.82 ± 0.28^a^	7.57 ± 0.23^a^
BC2		6.04 ± 0.33^b^

## Discussion

Microbial and non-microbial biostimulants play a primary role in sustainable agriculture by enhancing plant growth, nutrient use efficiency, and stress resilience, potentially reducing reliance on agrochemical inputs. However, the effective application of biostimulants requires the development of novel biobased formulations capable of preserving activity and cell vitality during production and storage, while ensuring efficient release of the encapsulated compounds or viable microbes to the crop (Pereira et al. 2023). Biobased carriers formulated with cellulose, agarose, chitosan, collagen, xanthan, and arabic gum have shown promising results in terms of enhanced biostimulant delivery and PGP effects in many model crops (Khan et al. 2023). The development of biobased carriers derived from food waste and by-products offers nevertheless a circular alternative that potentially better aligns with sustainable development goals. In this work, we evaluated three biostimulants, including a phytoextract obtained from lettuce waste and two PGP bacterial strains, *Bacillus* sp. LR01 and *Rhizobium* sp. GR12, for their suitability in two novel biobased carriers (BC1 and BC2) based on cellulose and pectin, both biopolymers that can be obtained from vegetable waste. Our findings demonstrated both the potential and the limitations of these carriers, compared to conventional delivery strategies such as liquid biostimulants and encapsulation in alginate beads.

The two bacterial strains were selected for this study from a previous collection of rhizosphere isolates based on demonstrated plant beneficial traits including increase in shoot height, nitrogen accumulation, and root biomass (Carullo et al. 2024; Vergani et al. 2024). Their ability to withstand environmental stressors was assessed in this work as a prerequisite for strain survival during encapsulation in the biopolymers, desiccation, and storage at room temperature. While *Rhizobium* sp. GR12 was the only strain showing psychrotolerance, being capable of growing at 4 °C, the spore-forming *Bacillus* sp. LR01 demonstrated greater tolerance to osmotic and saline stress, moderate high temperature, and acid pH. In addition, only *Bacillus* sp. LR01 exhibited polysaccharidase activities (pectinase, amylase, agarase, alginase), further suggesting its suitability for incorporation into dry polymer-based formulations designed for soil application, which may also be relevant in increasing the biodegradability of the carrier: enzymatic degradation of the biopolymer matrix may facilitate gradual release of bacteria and, at the same time, provide a carbon source to support cell revitalization and early colonization (Valdivia-Rivera et al. 2021). *Bacillus* sp. LR01 proved more robust than *Rhizobium* sp. GR12 in the development of biobased dry formulations for bacterial delivery, likely due to its spore production, reinforcing the value of the *Bacillus* genus in agro-biotech applications (Etesami et al. 2023). When inoculated on *L.* *sativa* var. expertise and *S. lycopersicum* var. Rio grande as aqueous suspensions, both strains confirmed the ability to establish themselves in the rhizosphere under greenhouse conditions, although with different efficiency and behaviours. *Bacillus* sp. LR01 established in the lettuce plant rhizosphere with higher cell concentration in comparison with *Rhizobium* sp. GR12, whereas in tomato plantlets, the opposite trend was observed, highlighting a degree of host-bacterial specificity. The two PGPB showed distinct root colonization strategies in vitro as assessed exploiting GFP-labelled mutants. *Rhizobium* sp. GR12 exhibited endophytic behaviour in both lettuce and tomato, with preferred colonization hubs on root hairs and epidermal cell wall junctions. This colonization progressed to the cortex and xylem vessels, with the latter suggesting potential transport throughout the root system and possibly to above-ground organs, a dynamic observed in other non-nodulating endophytic PGPB (Pallucchini et al. 2024). In contrast, *Bacillus* sp. LR01 displayed an exclusively epiphytic colonization pattern, distributing evenly along the epidermis and root hairs with no apparent preference for specific niches or progression over time, indicating a consistent surface-level association along the entire root length. No localization on lateral root emergence sites was observed either, ruling out endophytic invasion via the crack-entry mechanism, which is consistent with the well-known colonization mechanism of *Bacillus* sp. which involves surface-associated biofilm formation (Engelhardt et al. 2024).

A novel phytoextract (PE), derived from fresh-cut vegetable waste (mainly lettuce) via aqueous maceration, was also evaluated as a non-microbial biostimulant. The composition of the PE comprised readily available mineral nutrients (notably high in K), nitrogen, and organic carbon. Phytoextract-based biostimulants obtained via aqueous maceration—a simple extraction technique in which plant material is soaked in water to allow passive diffusion of soluble compounds—represent low-cost tools that align with circular economy and low-impact production principles, thanks to the minimal energy input required (Sudipto Debnath 2024). Although the exact mechanisms by which phytoextracts exert their PGP effect remain to be fully elucidated, they were shown to enhance physiological performance by stimulating nitrate assimilation, electron transport, and secondary metabolite accumulation (Franzoni et al. 2021). The presence of phytohormones (such as gibberellins, indole-3-acetic acid, and isopentenyladenosine) and diverse phenolic compounds previously reported in other cold-extracted vegetable extracts and known to influence carbon and nitrogen metabolism supports the hypothesis that aqueous phytoextracts can modulate key metabolic pathways, potentially enhancing the activity of enzymes involved in glycolysis, the Krebs cycle, and nitrate assimilation, contributing to the overall biostimulant effect (Ertani et al. 2016). For the delivery of the three biostimulants, the BC1 biomaterial based on microfibrillated cellulose, pectin, and glycerol was selected among a wider panel of formulations (Carullo et al. 2024). In addition, a second formulation, named BC2, was developed by incorporating silica into BC1 to enhance mechanical strength and reduce water solubility through increased matrix rigidity and cross-linking density. Both liquid formulations can be cast and dried at 37 °C to obtain semirigid, plastic-like films, with BC1 exhibiting higher flexibility and BC2 showing increased brittleness. Notably, all major components of these biopolymers—cellulose, pectin, and glycerol—can be sustainably recovered from agri-food waste streams, enabling a circular approach to carrier production (Iñiguez-Moreno et al. 2024). Although conventional pectin extraction can entail a non-negligible environmental impact due to high energy and acid consumption, emerging green extraction technologies now allow its recovery from fruit residues with markedly reduced environmental burdens (Riyamol et al. 2023).

The two biostimulant bacterial strains were incorporated in BC1 and BC2 to evaluate cell survival during the biomaterial formulation, desiccation, and storage at room temperature. *Rhizobium* sp. GR12 completely lost viability upon solidification in either BC formulation films, highlighting a limitation of dry-film-based carriers for non-spore-forming vegetative cells. This effect may be attributed to desiccation stress, which is well known to reduce bacterial viability (Greffe & Michiels 2020), or to a drop in pH following biopolymer dissolution in water, since pectin can exhibit acidic properties when solubilized due to its high content of galacturonic acid, potentially creating unfavourable conditions for sensitive microbial strains (Riyamol et al. 2023). Consequently, *Rhizobium* sp. GR12 was encapsulated in alginate beads, a well-established biocarrier used here as a benchmark for comparison with the novel BC biobased carriers developed in this study. The process of encapsulation in alginate beads resulted in a one-order-of-magnitude reduction in viable cells and supported the survival of *Rhizobium* sp. GR12 with no further loss in viability for up to 4 weeks at room temperature, and for as long as 6 months at 4 °C (data not shown). Alginate is a widely used matrix for biostimulant encapsulation due to its non-toxic nature and its property to slowly release bacteria into the environment (Bashan et al. 2002), potentially maintaining cell viability for as long as 14 years (Bashan & Gonzalez 1999) and ensuring bacterial delivery to promote crop growth (Liao et al. 2026). However, like other conventional biomaterials used for microbial delivery, alginate depends on a dedicated industrial production chain, as it is primarily extracted from brown seaweeds rather than being derived from waste streams (Ponce et al. 2025), increasing its environmental impact (Bularz et al. 2022). In contrast to *Rhizobium* sp. GR12, the incorporation of *Bacillus* sp. LR01 into both BC carriers resulted in the strain survival, most likely due to the highest tolerance of this strain to osmotic, saline, and pH stress and/or to the production of spores, which are known to withstand desiccation and extreme stress conditions (Cho & Chung 2020). Aiming to minimize experimental variability, *Bacillus* sp. LR01 was incorporated into the biobased carriers in the form of spores and showed a one-order-of-magnitude viability loss in the BC1 material and an over two-orders-of-magnitude decrease in BC2 after the desiccation process, with no further viability decrease after 4 weeks at room temperature. When tested for biodegradability, however, the BC1 biomaterial demonstrated limited stability in soil, with complete dissolution after 2 to 4 days, which would lead to the immediate, rather than gradual, release of the embedded biostimulants. In contrast, the silica-enriched BC2 formulation retained some structural integrity for up to 1 month. Therefore, despite the decrease in spore viability caused by BC2, this biomaterial was selected for subsequent greenhouse experiments as the carrier matrix for the incorporation and delivery of both *Bacillus* sp. LR01 spores and PE biostimulant, as a delayed and gradual release was considered a positive factor for sustained biostimulant effect over time (Balla et al. 2022).

The application of the three biostimulants—*Bacillus* sp. LR01, *Rhizobium* sp. GR12, and the PE—was evaluated for promoting lettuce growth under greenhouse conditions, comparing direct aqueous inoculation to biobased carrier delivery via alginate beads or BC2 films. The inclusion of both carrier-free and carrier-only controls allowed to disentangle the intrinsic effect of each biostimulant, the effect of carrier-based delivery, and potential carrier-specific effects. When applied as a liquid suspension directly to the soil after seed sowing, all three biostimulants significantly improved plant growth compared to the untreated negative controls following percent control transformation: shoot fresh weight increased by +21.5% (PE), +29.9% (*Bacillus* sp. LR01), and +24.5% (*Rhizobium* sp. GR12) compared to the control. Shoot height was also significantly increased by PE (+23.9%) and LR01 (+32.9%). Chlorophyll content remained largely unaffected, suggesting a genuine stimulation of growth rather than stress-induced physiological shifts (Guidi et al. 2019). Notably, liquid PE application reduced the leaf phenolic index (−14.5%), suggesting alleviation of basal stress or a shift away from carbon allocation to secondary metabolism (Rao & Zheng 2025), consistent with a growth-promoting, low-stress state typical of effective biostimulant action (Franzoni et al. 2022). In contrast, the application of the biostimulants through biobased carriers applied to the soil during seed sowing generally resulted in the reduction, and in some cases the loss, of such statistically significant positive effects, despite maintaining bacterial viability and allowing rhizosphere colonization. Encapsulation of *Rhizobium* sp. GR12 in alginate beads, notably, resulted in a marked improvement in rhizosphere colonization, reaching almost two-orders-of-magnitude higher CFU counts than what obtained with the liquid inoculum, thus confirming the protective and sustained-release properties of the alginate matrix. However, this increased rhizosphere colonization did not translate into improved plant growth performance; instead, the significant shoot biomass increase seen with aqueous application was lost. The incorporation of *Bacillus* sp. LR01 into alginate beads only marginally increased its colonization rate compared to the liquid inoculum, but it also nullified the significance of both shoot weight and height increase observed with the liquid inocula. However, the delivery of *Bacillus* sp. LR01 via alginate beads elicited a decrease in phenolic index and an increase in chlorophyll, with a consequent rise in the nitrogen-flavonol index, an optical parameter that reflects the balance between leaf nitrogen status and flavonol accumulation. Since flavonols typically increase under stress while chlorophyll declines, the combination of lower phenolic index and higher chlorophyll is consistent with a low-stress, growth-promoting physiological state (Franzoni et al. 2022; Guidi et al. 2019; Rao & Zheng 2025) that may sustain plant growth on a later growth stage. As expected, alginate beads tested without any embedded biostimulants showed no statistically significant effect on lettuce growth and physiology. The observed decoupling between colonization rate and plant response is consistent with the slow release of bacteria from alginate matrices, which can cause inoculants to miss the critical early window for root signalling and colonization (Bashan 1986). Moreover, encapsulated bacteria frequently exhibit reduced initial metabolic activity, with a proportion of cells entering a lag phase or dormant state before resuming growth (John et al. 2011), in contrast to water-inoculated cells that are applied during their exponential phase. Spatial heterogeneity associated with alginate bead distribution may further restrict early microbial contact with the root system, whereas aqueous inoculation ensures more uniform rhizosphere coverage (Bashan et al. 2014). Alternatively, the increased microbial abundance in the rhizosphere induced by the alginate carrier may enhance plant immune activation, as beneficial microbes are known to trigger basal and induced systemic responses even in the absence of pathogens (Pieterse et al. 2014). All these factors are particularly relevant during early seedling establishment, when plant responsiveness to microbial signals is higher, and therefore may have prevented effective engagement with early root signalling processes that are essential for growth promotion. It is important to note that in this study, the PGP effect of biomaterial-delivered inoculants was assessed at the seedling stage, when plants had reached a size appropriate for transplanting, a stage relevant for product application within horticultural nurseries. Future work should extend these evaluations to the stage of commercial harvest, as PGPB delivered through biocarriers may exert delayed or cumulative effects during later phases of plant development.

Similarly to *Rhizobium* sp. GR12 delivered in alginate, the BC2 carrier also enabled successful soil establishment and rhizosphere colonization of *Bacillus* sp. LR01 spores, although at an order of magnitude lower abundance compared with the liquid inoculum or alginate beads. These results indicate that the BC2 matrix did not impair spore germination and rhizosphere competence, supporting the feasibility of this formulation for spore-forming strains. However, as observed for alginate delivery, BC2 nullified the statistically significant positive effects on shoot weight and height given by the direct liquid spore inoculation. Despite the negative effects observed on plant biomass, *Bacillus* sp. LR01 exhibited a potentially beneficial response when delivered via BC2, as reflected by a reduction in phenolic index (−15.6%), mirroring the trend observed with alginate beads and contrasting with the direct liquid inoculum. When the PE was incorporated into BC2, the negative impact was even more pronounced, with shoot weight significantly reduced relative to the uninoculated control. BC2-delivered PE also increased the flavonol index by 27.8%, similarly to the uninoculated BC2 material that induced a rise in flavonols (+22.2%) and a corresponding decrease in the nitrogen-flavonol index (−2.4%), indicating a stress-associated metabolic shift. This response may arise from the release of BC2 degradation products into the root zone, which can alter rhizosphere chemistry as previously described for biodegradable plastics (Withana et al. 2025), including potential acidification that may negatively affect plant performance. While the increased phenolic accumulation observed in treated plants is consistent with this hypothesis, the specific mechanisms in this system remain to be clarified. Literature reports that biopolymer degradation products can also trigger mild plant defence activation, alter local oxygen availability—an important determinant of root-associated microbial activity—and modify nutrient diffusion patterns, any of which could contribute to the observed responses and potentially disrupt plant–microbe signalling (Thapliyal & Khan 2024; Withana et al. 2025). Additional factors, such as interactions between the BC2 matrix and root exudates or hormone dynamics, have also been proposed for other biopolymers (Mohammadian et al. 2024), but further work is required to determine whether similar processes occur. Finally, biopolymer-induced modulation of the soil microbiota may contribute to the impaired plant development (Li et al. 2025), as changes in microbial community structure are known to strongly influence plant growth and stress responses. However, further characterization of the chemical and mechanical degradation of BC2 in soil and its impact on plant physiology will be required to validate such hypothesis and provide mechanistic explanation of the observed effects, providing information for the optimization of its agronomic compatibility. The contrasting physiological responses observed in this study indicate that biobased carriers can impact plant growth performance in spite of biostimulants efficiency and differentially influence plant–microbe interactions in a way that is independent from bacterial viability and successful establishment in soil. While main concerns regarding biostimulant delivery systems often regard their effectiveness in protecting microbial viability and improving plant colonization, our results suggest that, even when those properties are achieved, attention should focus on the carrier-driven plant physiological modulation. Moreover, our findings indicate that the same carrier may elicit distinct metabolic and growth outcomes depending on the biostimulant strain encapsulated, underscoring that carrier selection must be tailored to the biological and physiological characteristics of the specific inoculated microorganism rather than assumed to be universally compatible.

## Conclusions

Carrier materials play a pivotal role in agriculture by ensuring controlled release and improving the synchronization of fertilizer or biostimulant availability with crop demand. In this context, a circular economy approach that exploits biopolymers extracted from agri-food waste and by-products can be regarded as a smart and sustainable substitute for plastic-derived materials. However, the findings of this study indicate that a biobased origin alone does not guarantee the suitability of a material as a biostimulant carrier. Our results reinforce the importance of selecting carriers that not only support microbial viability and controlled biostimulant release, but are also compatible with the whole plant–soil system. Future efforts should focus on fine-tuning the chemical composition of different formulations of these novel biobased materials and on the molecular understanding of their degradation profile to elucidate both plant physiology modulation and interaction with the soil microbiome. Furthermore, assessing the influence of biopolymer-delivered biostimulants across the entire plant life cycle is essential to determine whether the long-term effects on plant growth align with the physiological responses observed in this study at the transplant stage.

## Supplementary Information

Below is the link to the electronic supplementary material.

ESM 1 (PDF 663 KB)

## Data Availability

All data supporting the findings of this study are available within the paper and its Supplementary Information. The bacterial strains used in this work are available at University of Milan, Biotechnology and Environmental Microbiology Laboratory, upon request to francesca.mapelli@unimi.it.
