# Supplementary material for: Evaluation of biobased carriers derived from agri-food waste for biostimulants delivery in horticulture
Source: Appl Microbiol Biotechnol. 2026 May 28;110:228. doi: 10.1007/s00253-026-13886-y (PMC13407616; doi:10.1007/s00253-026-13886-y)
Supplement: Supplementary file 1 [file 253_2026_13886_MOESM1_ESM.pdf]

Evaluation of biobased carriers derived from agri-food waste for biostimulants delivery in horticulture

Michele Pallucchini[1], Francesca Mapelli[1], Giulia Franzoni[2,3], Daniele Carullo[1], Joa Patania[1], Nicolò De Pizzol[2], Antonio Ferrante[2,4], Stefano Farris[1], Lorenzo Vergani[1*], Sara Borin[1]

[1] Department of Food, Environmental and Nutritional Sciences, University of Milan, Milan, Italy
[2] Department of Agricultural and Environmental Sciences, University of Milan, Milan, Italy
[3] Department of Earth and Environmental Sciences, University of Pavia, Pavia, Italy
[4] Institute of Crop Science, Sant'Anna School of Advanced Studies, Pisa, Italy
[*]Corresponding author: lorenzo.vergani@unimi.it

Supplementary Tables

Table S1. Summary of the ten treatments applied to lettuce plants in the greenhouse trials described in the materials and methods section. For each treatment we indicated the identification code as reported in the Figure 4 and S3.

	Treatment	Code
1	*Bacillus* sp. LR01 spores embedded in BC2 film	BC2-LR01
2	*Bacillus* sp. LR01 spores encapsulated in alginate beads	A-LR01
3	*Rhizobium sp.* GR12 cells encapsulated in alginate beads	A-GR12
4	Phytoextract incorporated into BC2 film	BC2-PE
5	*Rhizobium sp.* GR12 cells as aqueous suspension	GR12
6	*Bacillus* sp. LR01 spores as aqueous suspension	LR01
7	Phytoextract as aqueous solution	PE
8	Water (negative control)	NC
9	Uninoculated BC2 film	BC2
10	Uninoculated alginate beads	A

Supplementary Figures

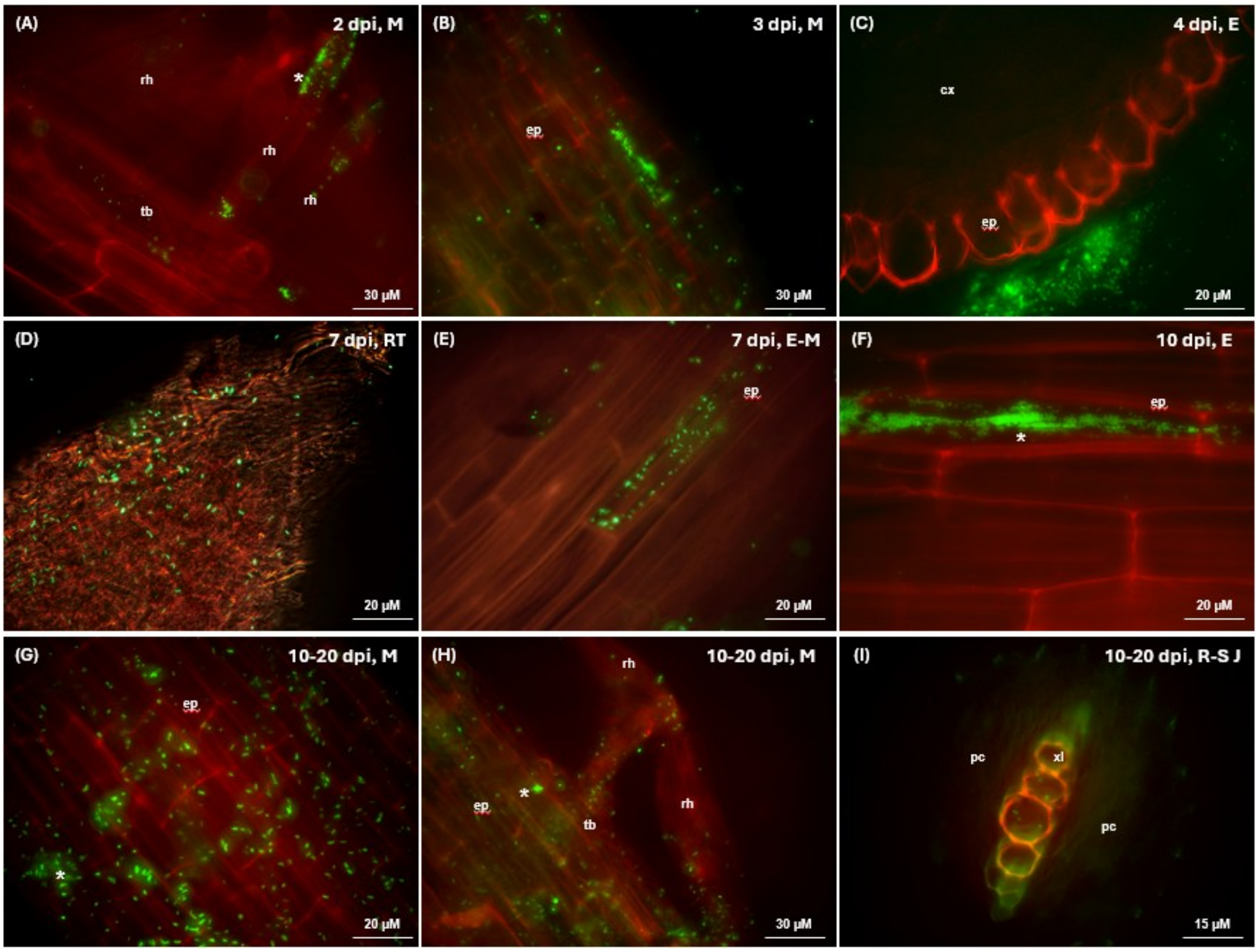

Figure S1. Colonization of tomato root by fluorescently tagged *Rhizobium* sp. GR12 on MS agar, 1 to 20 dpi. Fluorescence from bacterial *gfpmut3** and from plant tissues dyed with rhodamine B is visualised as green and red signals, respectively, through fluorescence microscopy. In the top right corner of each picture, the timepoint of observation (dpi) and anatomical zone of the root are indicated (E = elongation; M = maturation; RT = root tip; R-S J = root-shoot junction). (A) Epiphytic colonization of root hairs and trichoblasts by bacterial aggregates (white stars) during early colonization stages. (B-C) Epiphytic biofilm (white stars) and individual bacterial cells colonising the root epidermis. (D) Root tip showing colonization by interspersed bacterial cells. (E-F) Endophytic colonisation of root epidermal cells. (G-H) Extensive colonization of epidermal cells and root hairs along the elongation and maturation zones by both individual cells and bacterial aggregates (white stars) during later interaction stages. (H) Root cross sections showing endophytic colonization of xylem vessels. ep, epidermis; rh, root hair; pc, procambium; tb, trichoblast.

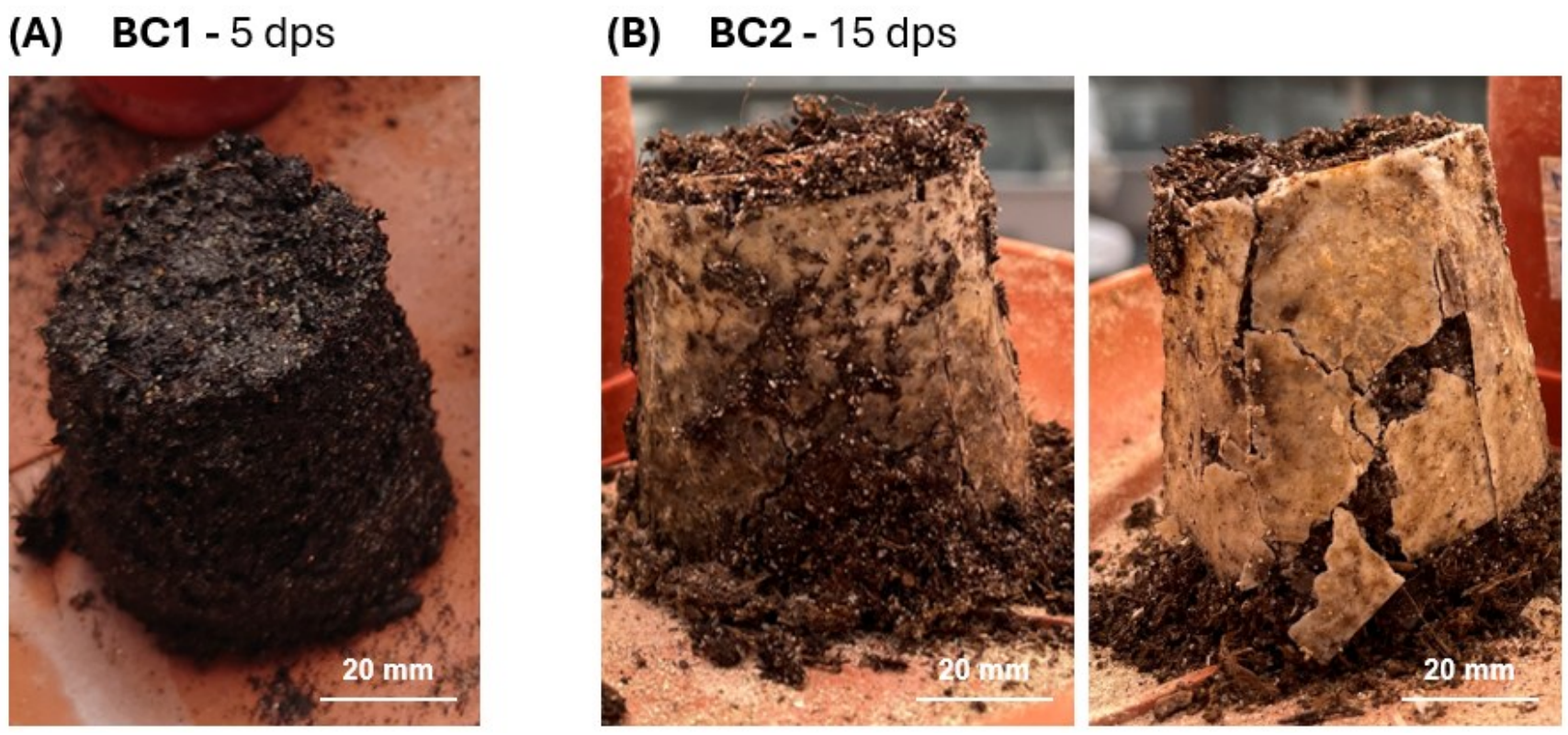

Figure S2. Biodegradation of uninoculated BC1 (A) and BC2 (B) films 5 to 15 days post-sowing (dps) in germination soil without plants.

Shoot weight (g)

		NC	BC2	A	PE	BC2 - PE	LR01	BC2 - LR01	A - LR01	GR12	A - GR12
Trial 1	AVG	2.78^{bc}	2.34^{c}		3.60^{a}	2.50^{c}	3.66^{a}	2.75^{c}	2.68^{c}	3.40^{ab}	2.61^{c}
	SD	0.29	0.28		0.33	0.65	0.46	0.59	0.42	0.39	0.18
Trial 2	AVG	5.85^{bc}	6.37^{ab}	6.09^{bc}	6.63^{ab}	4.16^{cd}	7.49^{a}	4.93^{cd}		7.39^{a}	5.67^{bc}
	SD	0.50	0.70	0.95	0.46	1.14	0.67	1.09		0.87	0.78
Trial 3	AVG	3.23		3.60	3.49		4.56		4.00	4.26	3.72
	SD	1.29		1.16	1.37		1.81		0.66	1.60	1.32

Shoot height (cm)

		NC	BC2	A	PE	BC2 - PE	LR01	BC2 - LR01	A - LR01	GR12	A - GR12
Trial 1	AVG	8.3^{cde}	7.43^{e}		9.73^{ab}	8.45^{cd}	10.02^{a}	8.95^{bc}	8.09^{cde}	8.2^{cde}	7.55^{de}
	SD	0.45	0.58		0.42	0.82	0.90	0.52	0.65	0.37	0.52
Trial 2	AVG	9.34^{ab}	8.93^{abc}	9.1^{abc}	9^{abc}	8.18^{c}	9.71^{a}	9.01^{abc}		9.69^{a}	8.59^{bc}
	SD	0.53	0.69	0.61	0.43	0.71	0.66	0.73		0.57	0.60
Trial 3	AVG	8.50		9.03	8.59		9.49		8.96	7.44	8.74
	SD	1.63		0.96	1.14		1.09		0.90	2.79	0.86

Chlorophyll a + b (µg/mg)

		NC	BC2	A	PE	BC2 - PE	LR01	BC2 - LR01	A - LR01	GR12	A - GR12
Trial 1	AVG	1.38^{ab}	1.44^{ab}		1.62^{a}	1.36^{ab}	1.45^{ab}	1.49^{ab}	1.2^{b}	1.38^{ab}	1.36^{ab}
	SD	0.18	0.18		0.16	0.14	0.18	0.08	0.09	0.09	0.12
Trial 2	AVG	1.06^{a}	0.93^{ab}	0.97^{ab}	1.12^{a}	0.93^{ab}	0.84^{ab}	0.79^{b}		0.93^{ab}	0.95^{ab}
	SD	0.09	0.27	0.09	0.23	0.12	0.09	0.17		0.11	0.26
Trial 3	AVG										
	SD										

Phenolic Index (ABS 320 nm/g)

		NC	BC2	A	PE	BC2 - PE	LR01	BC2 - LR01	A - LR01	GR12	A - GR12
Trial 1	AVG	31.26^{ab}	31.18^{ab}		24.26^{c}	36.65^{a}	31.14^{ab}	28.34^{bc}	26.01^{bc}	27.44^{bc}	25.77^{bc}
	SD	2.18	1.30		1.64	4.14	4.02	3.97	1.11	1.12	2.71
Trial 2	AVG	5.32	4.10	5.62	5.97	4.50	5.42	4.16		5.80	5.31
	SD	0.22	0.44	0.86	1.82	0.55	0.96	0.51		1.40	0.86
Trial 3	AVG										
	SD										

Chlorophyll Content Index (MPM-100)

		NC	BC2	A	PE	BC2 - PE	LR01	BC2 - LR01	A - LR01	GR12	A - GR12
Trial 1	AVG	0.52^{cd}	0.52^{cd}		0.53^{c}	0.52^{cd}	0.50^{cd}	0.43^{d}	0.68^{a}	0.56^{bc}	0.64^{ab}
	SD	0.07	0.08		0.09	0.09	0.13	0.07	0.07	0.09	0.10
Trial 2	AVG	0.53^{ab}	0.46^{b}	0.57^{a}	0.49^{ab}	0.47^{ab}	0.51^{ab}	0.48^{ab}		0.56^{ab}	0.49^{ab}
	SD	0.10	0.14	0.10	0.14	0.10	0.12	0.09		0.17	0.12
Trial 3	AVG	0.49^{ab}		0.46^{ab}	0.52^{ab}		0.48^{ab}		0.56^{a}	0.43^{b}	0.51^{ab}
	SD	0.08		0.08	0.21		0.08		0.15	0.08	0.12

Flavonol Content Index (MPM-100)

		NC	BC2	A	PE	BC2 - PE	LR01	BC2 - LR01	A - LR01	GR12	A - GR12
Trial 1	AVG	0.18^{bcd}	0.26^{a}		0.15^{d}	0.19^{bcd}	0.16^{cd}	0.19^{bc}	0.21^{b}	0.20^{b}	0.21^{b}
	SD	0.03	0.06		0.03	0.05	0.03	0.04	0.03	0.03	0.02
Trial 2	AVG	0.23^{bc}	0.25^{bc}	0.26^{b}	0.26^{bc}	0.33^{a}	0.21^{bc}	0.25^{bc}		0.20^{c}	0.25^{bc}
	SD	0.05	0.05	0.07	0.07	0.07	0.06	0.08		0.06	0.08
Trial 3	AVG	0.21		0.18	0.20		0.18		0.17	0.19	0.20
	SD	0.05		0.06	0.03		0.05		0.06	0.04	0.05

Nitrogen Flavonol Index (MPM-100)

		NC	BC2	A	PE	BC2 - PE	LR01	BC2 - LR01	A - LR01	GR12	A - GR12
Trial 1	AVG	2.99^{ab}	2.15^{c}		3.67^{a}	3.00^{ab}	3.38^{a}	2.43^{bc}	3.34^{a}	2.93^{abc}	3.11^{ab}
	SD	0.51	0.72		1.02	0.99	1.14	0.70	0.75	0.56	0.66
Trial 2	AVG	2.48^{ab}	1.94^{ab}	2.25^{ab}	2.39^{ab}	1.45^{c}	2.69^{a}	2.05^{abc}		2.92^{a}	2.26^{abc}
	SD	0.79	0.92	0.68	1.00	0.60	0.98	0.81		1.19	0.96
Trial 3	AVG	2.16^{b}		3.03^{ab}	2.70^{ab}		2.89^{ab}		3.57^{a}	2.52^{ab}	2.72^{ab}
	SD	0.96		1.32	1.16		1.19		1.31	0.88	0.68

Figure S3. Plant growth parameters of inoculated lettuce plants grown under greenhouse conditions. All measurements were taken 25 dpi. Values from three independent experiments are reported as mean (AVG) ± standard deviation (SD) for each treatment and trial (sample size ≥5). Different letters (blue exponents of AVG values) indicate statistically significant differences among treatments within the same trial. Absence of numerical values indicates treatments not included or parameters that were not evaluated due to experimental constrains. Statistical significance was assessed using Shapiro-Wilk tests for normality followed by One-way ANOVA with Tukey's HSD or Kruskal-Wallis test with Dunn's post hoc comparison when assumptions were not met ($P \leq 0.05$). BC2 = uninoculated BC2 film; A = uninoculated alginate beads; PE = phytoextract; BC2-PE = phytoextract incorporated into BC2 film; LR01 = aqueous inoculum of *Bacillus* sp. LR01 spores; BC2-LR01 = *Bacillus* sp. LR01 spores incorporated into BC2 film; A-LR01 = *Bacillus* sp. LR01 spores encapsulated into alginate beads; GR12 = aqueous inoculum of *Rhizobium* sp. GR12 cells; A-GR12 = *Rhizobium* sp. GR12 encapsulated into alginate beads.

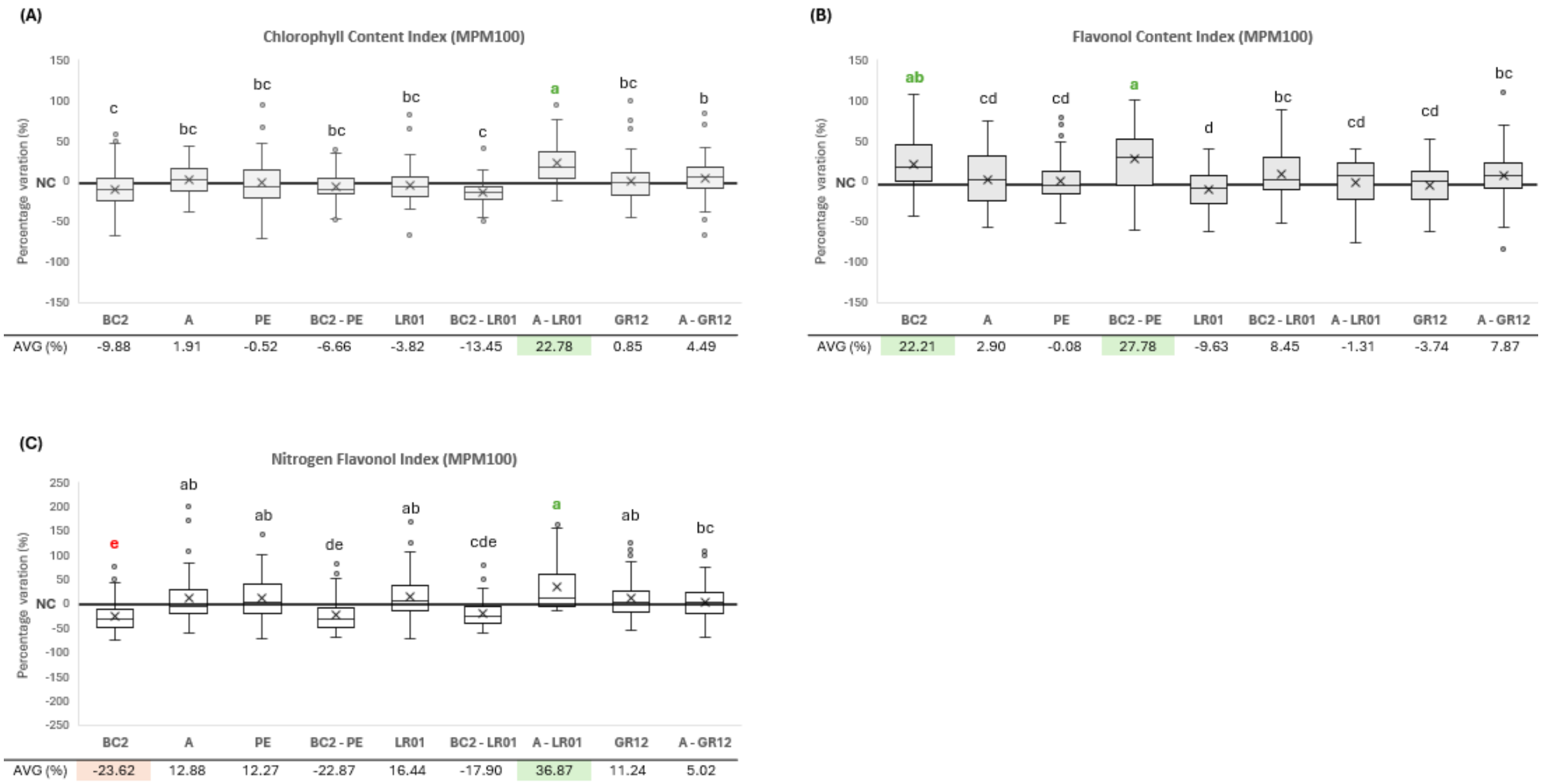

Figure S4. A-C) Percentage variation of plant growth parameters in inoculated lettuce plants grown under greenhouse conditions. All measurements were taken 25 dpi. All values represent the percentage variation of each treatment relative to the mean of the uninoculated control (NC) within each experiment. The central horizontal line at 0% corresponds to the normalized NC baseline. Data from three independent experiments were pooled after within-experiment normalization. Statistical significance was assessed using Shapiro-Wilk tests for normality followed by One-way ANOVA with Tukey's HSD or Kruskal-Wallis test with Dunn's post hoc comparison when assumptions were not met ($P \leq 0.05$). Green bold labels indicate significant increases relative to NC, whereas red bold labels indicate significant decreases. Numerical values indicating the mean percentage variation for each treatment are reported below the boxplots and shaded in green for significant % increase or in red for significant % decrease. A) Shoot weight. B) Shoot height. C) Chlorophyll a + b content (destructive measurements). D) Phenolic index (destructive measurements). BC2 = uninoculated BC2 film; A = uninoculated alginate beads; PE = phytoextract; BC2-PE = phytoextract incorporated into BC2 film; LR01 = aqueous inoculum of *Bacillus* sp. LR01 spores; BC2-LR01 = *Bacillus* sp. LR01 spores incorporated into BC2 film; A-LR01 = *Bacillus* sp. LR01 spores encapsulated into alginate beads; GR12 = aqueous inoculum of *Rhizobium* sp. GR12 cells; A-GR12 = *Rhizobium* sp. GR12 encapsulated into alginate beads.
